# Neurography - a new look at the peripheral nervous system

**DOI:** 10.1590/0100-3984.2017.50.4e3

**Published:** 2017

**Authors:** Fernanda C. Rueda Lopes

**Affiliations:** 1 Adjunct Professor of Radiology at the Universidade Federal do Rio de Janeiro (UFRJ), Rio de Janeiro, RJ, Brazil. E-mail: frueda81@hotmail.com

The advent of magnetic resonance imaging (MRI) allowed a significant advance in the understanding and evaluation of the central nervous system. However, the peripheral nervous system (PNS)-nerves, ganglia, and roots-continued to be considered "difficult to assess" by radiologists^(1)^. The PNS has been studied through invasive tests, such as electrodiagnostic studies and nerve biopsies, which cause patient discomfort^(2,3)^. In this context, the development of magnetic fields that were more powerful, which allowed the acquisition of fine sequences with high resolution, made it possible to study the PNS by MR neurography, the first of such studies being published in 1993^(1)^.

MR neurography is a technique involving regional MRI that allows evaluation of the longitudinal axis of the peripheral nerves, with the possibility of multiplanar reconstruction^(2)^. The basic structural sequences for MR neurography, which evaluate the signal strength of the images^(1)^, include fat suppression sequences, typically with inversion recovery or even the Dixon technique^(2,4)^, with high (0.2-0.5 × 0.2-0.5 mm) spatial resolution^(4)^, isotropic sequences, and sequences with three-dimensional acquisition for multiplanar reformatting^(2)^. The functional quantitative analysis, which includes the evaluation of the directionality of the data^(1)^, is based on diffusion tensor imaging (DTI). For that purpose, the 3.0 T devices (with high signal-to-noise ratio) present better performance^(2)^. Additional sequences can be used, such as T2-weighted fat-suppressed sequences of the affected limb, for the evaluation of muscle edema/denervation^(2)^, and intravenous contrastenhanced T1-weighted fat-suppressed sequences, for the evaluation of neural tumors^(5)^. The tractography reconstruction based on the raw DTI data is more feasible in the lumbosacral plexus (given that the data are acquired in the isocenter of the magnet) than in the brachial plexus and peripheral nerves^(2)^. The study of the brachial plexus can be limited because of the interfaces between the various body tissues and air, making it difficult to fully suppress fat^(5)^.

The indication for MR neurography will define its length and duration. MR neurography is more efficacious than are electrodiagnostic studies in the evaluation of deep structures^(1)^, the main indications being related to inflammatory/genetic/immune-mediated diseases of the peripheral nerves, neural tumors, or posttraumatic alterations^(3,5)^. If the neuropathy is extensive, as in cases of Charcot-Marie-Tooth disease, MR neurography can cover all the nerve trunks, whereas directed sequences can be employed in the evaluation of localized lesions, such as a sciatic compression caused by lumbar disc herniation. However, the minimum time required to perform the examination is 50 min.

The high spatial resolution of the inversion recovery sequence allows the identification of the nerve fascicles^(4)^. In normal nerves, the signal intensity is highest in the dorsal root ganglion, decreasing along the trajectory of the nerve, which tapers distally^(2)^. The initial reading of an MR neurography scan consists of assessing the persistence of the higher-intensity signal and areas of thickening along the nerve^(2)^. The identification of rupture is useful in post-traumatic neuropathy^(6)^. The affected nerve presents high signal intensity in T2-weighted sequences, due to prolongation of the T2 relaxation time, based on the dynamics of the endoneurial fluid^(1)^, although this finding is not very specific for the etiology and severity of the lesion^(6)^. The test should be complemented with DTI, which, through its fractional anisotropy parameter, allows the density of the fibers to be evaluated in a manner quite similar way to that of the histopathology. Reduced fractional anisotropy values suggest facilitation of diffusion along the nerve, representing axonal loss or demyelination, as assessed in patients with type I diabetes and polyneuropathy^(4)^.

A pictorial essay published in the previous issue of **Radiologia Brasileira** illustrates quite well cases in which MR neurography was employed for the evaluation of sciatic nerve damage^(7)^. As previously mentioned, the lumbosacral plexus and consequently the sciatic nerve are best evaluated by MR neurography, and the representation of the diseases highlighted here can be replicated in the remainder of the PNS. We also stress the importance of additional sequences for diagnostic complementation, such as intravenous contrast-enhanced sequences and sequences designed to evaluate muscle denervation.

MR neurography is being widely developed and will undoubtedly become an important radiological tool for the evaluation of the PNS.

## References

[1] Filler A (2009). Magnetic resonance neurography and diffusion tensor imaging:
origins, history, and clinical impact of the first 50,000 cases with an
assessment of efficacy and utility in a prospective 5000 patient study
group. Neurosurgery.

[2] Chhabra A, Carrino JA, Farahani SJ (2016). Whole-body MR neurography: prospective feasibility study in
polyneuropathy and Charcot-Marie-Tooth disease. J Magn Reson Imaging.

[3] Wang KC, Salunkhe AR, Morrison JJ (2015). Ontology-based image navigation: exploring 3.0-T MR neurography
of the brachial plexus using AIM and RadLex. Radiographics.

[4] Vaeggemose M, Pham M, Ringgaard S (2017). Diffusion tensor imaging MR neurography for the detection of
polyneuropathy in type 1 diabetes. J Magn Reson Imaging.

[5] Grayev A, Reeder S, Hanna A (2016). Use of chemical shift encoded magnetic resonance imaging
(CSE-MRI) for high resolution fat-suppressed imaging of the brachial and
lumbosacral plexuses. Eur J Radiol.

[6] Boyer RB, Kelm ND, Riley DC (2015). 4.7-T diffusion tensor imaging of acute traumatic peripheral
nerve injury. Neurosurg Focus.

[7] Agnollitto PM, Chu MWK, Simão MN (2017). Sciatic neuropathy: findings on magnetic resonance
neurography. Radiol Bras.

